# Notes and News

**Published:** 1887-08-20

**Authors:** 


### Notes and News.

Collected and Communicated.

By the will of the late Sir Barrow Ellis, of Cromwell Road, University College Hospital receives £1,000 towards the general fund, and £1,000 towards the building extension fund.

A show of wild-flowers was held at the Waterloo Rooms, Glasgow, on Friday and Saturday. The proceeds are to be given to the Glasgow Children's Hospital.

By a majority of 215 in a total poll of 277, Mr. C. A. Sharp has been elected Honorary Surgeon to the Derbyshire General Infirmary. Mr. C. Vawdrey was the other candidate, and only had a diminished following because of Mr. Sharp's longer connection with the town and hospital.

Now that the Dublin Hospital system is in a fair way of being reformed, it may not be out of place to suggest that the voluntary principle of hospital establishment and maintenance might be tried on a more extended scale, not only without injury, but with very much advantage to the Dublin people. There is no particular politico-economic objection to the state endowment of hospitals, but a liberal measure of general benevolence indicates a healthy condition of public morality.

The nurses at the Radcliffe Hospital, Oxford, are sent out on private nursing engagements, and this is found to be so profitable, that although the patients at the hospital have been more numerous than usual, and the expenditure greater during the last quarter, the cost per bed for nursing has considerably diminished. Do the nurses like this change in their work from hospital to private nursing, and is the practice found to work well all round?

A resolution of condolence with Mrs. George Ward and her daughter, and of high appreciation of the valuable services of the late Mr. George Ward, was passed at the quarterly meeting of the Radcliffe Hospital Committee, Oxford, on Wednesday, the treasurer, Dr. Bright, Master of the University, being in the chair. Mr. Ward had been for many years Vice-Chairman of the Committee of Management.

The Totnes people have surprised the promoters of the bazaar on behalf of the local Cottage Hospital, both by their unexpected purchasing power and their disinterested benevolence. A total of nearly £300 was reached by sales and donations. Totnes clearly deserves to have a Cottage Hospital.

A total sum of £106,402 has been spent by the Glasgow Corporation on its hospitals at Belvidere. A separate hospital is provided for small-pox cases, all other infectious diseases, being treated in the larger building. The hospitals have been officially inspected, and are declared to be in a high state of efficiency.

The West London Hospital has established special departments for the treatment of diseases of the ear, skin, throat, etc. A special ophthalmic department has been in existence for some time. The patients in the neighbourhood much appreciate the saving of time and money thus secured to them. This is as it should be. Special hospitals should be as few as possible: special departments should be found at every general hospital.

The governors of the Hartlepools Hospital held a special meeting to select a house-surgeon on Tuesday, and unanimously appointed Mr. Morgan, M.R.C.S. At the same time they passed a resolution which requires some explanation. It reads thus: "that the house-surgeon do not absent himself from the hospital, except when the permission of the governors has been given, except on an emergency, when the medical staff can give him permission." Has the late house-surgeon been taking little trips to London or America on his own responsibility, that such a singular resolution should be passed? And is the new man to be so punished for his predecessor's sins, that he is never to be allowed to leave the hospital at all, except by special permission of the committee, or the medical staff? We know that according to Moses, who is supported by modern scientific evidence, the sins of the parents are visited on the children, even unto the third and fourth generation; but we do not think that either of these authorities contemplated such an extension of the principle as would include successive generations of house-surgeons.

THE following vacancies are announced this week, the amount of the salary being placed in each case after the name of the institution :—

**Resident Medical Officers.**

Bristol Royal Infirmary. Assistant Resident.—£80.
Denbighshire Infirmary. House Surgeon.—£15.
Female Lock Hospital, Harrow Road. Assistant House Surgeon.
General Hospital, Birmingham. House Governor.—£300.
General Infirmary, Leeds. Medical Officer.—£100.
Hulme Dispensary, Manchester. House Surgeon.—£130.
Liverpool Dispensaries. Assistant Surgeon.—£80.
Manchester Royal Infirmary. Resident Surgical Officer.—£150.
Consumption Hospital, Ventnor. Assistant Officer.

**Non-resident Medical Officers.**

Dueness, Sutherlandshire. Medical Officer.—£150.
Parochial Board of Pennygown and Torosay. Medical Officer.—£100.
Teignmouth Urban Sanitary District. Medical Officer of Health.
Weston-super-Mare Hospital and Provident Dispensary. Provident Officer.—£60.

**Matron.**

Manchester, Monsall Fever Hospital. Lady Superintendent.—£80.

**Trained Nurses.**

Bristol Royal Infirmary. One year's previous experience.—£16, increasing.
Erith Cottage Hospital.—£30. All found. Charge of Kitchen.
Kent and Canterbury Nurses' Institute. Two.

---

IN future there need be no delay in the transference of patients suffering from infectious diseases, to the fever hospitals provided by the Metropolitan Asylums Board. It has been resolved by the Local Government Board that the certificate of any registered practitioner shall be sufficient evidence of the nature of any given case, and to warrant its immediate removal to the hospital.

---

THE following men are now lying in St. Mary's Hospital, suffering from serious injuries sustained at the great fire at Whiteley's on Saturday, August 6th :—Charles E. Enticknapp, a police constable of the F division, who, while assisting to carry forward the hose of a manual engine, received a severe injury to his right foot; firemen Ambrose Lester and James Brown, who fell through a floor and ceiling among masses of burning timber and red-hot ruins, and both of whom were much burned about the face and hands, and otherwise injured; George E. Bettell, a carman, thigh broken and other injuries received, as he was lending a hand with the hose-pipe; Edwin Emms, a police-constable of the F division, scalp wound from a falling tile, left side cut, injury to back and right knee. The most serious case, however, is that of Charles Baldry, a coachman, who was a spectator when the wall fell in the early part of the fire. His right leg was so severely fractured that the limb had to be amputated. Thomas Lonsdale, the only other injured person whose case was severe enough to be kept in the hospital, had a fractured scapula, and head badly cut by falling *débris*. There were some score or more others brought in, in the course of Saturday evening, whose injuries were not sufficiently severe to necessitate their admission; amongst the earlier ones were Mr. Wrapson, engineer at the Baths, cut head; Mr. Osterfield, one of the parochial officers, cut fingers and bruised arm; the remainder were principally cuts and bruises about the head and body, received by the falling of stones and timber. At present there is no reason to anticipate that any of the injuries will terminate fatally.

THE following subjects have already been arranged for discussion at the evening meetings during the next session of The Hospitals Association, the gentlemen named having kindly consented to read papers :—

The Amelioration of the Condition of Midwives. By J. H. Aveling, Esq., M.D.
Contributions by Hospital Patients. By A. B. Burdett-Coutts, Esq., M.P.
The Relation of the Medical School to the Hospital. By T. Gilbart-Smith, Esq., M.D.
A National Pension Fund for Hospital Officials and Trained Nurses. By G. W. Potter, Esq., M.D.
The Origin, History, Work, and Present State of Metropolitan Lying-in Hospitals. By Thomas Ryan, Esq., Secretary of St. Mary's Hospital (late Secretary of Queen Charlotte's Lying-in Hospital).
Hospital Construction. By Keith D. Young, Esq., F.R.I.B.A.

Other papers are in course of arrangement, and members are reminded that the meetings are held on the second Wednesday of each month at 8 p.m.

---

ONLY one small-pox case in the whole of London during the hottest month in the year! We may be living on a slumbering volcano, but, in the meantime, we will congratulate ourselves on the gratifying fact. Have the antivaccinators anything to suggest by way of explanation? We do not ask sarcastically: we are for the fullest liberty of conscience; but we should like to know, as a matter of practical knowledge, what they think and say about the subject?

---

*The Globe* instructs its readers about nurses and nursing. Perhaps the amount of work expected from a nurse, and the length of time daily during which she is on duty, are a little exaggerated. The nurse's calling is undoubtedly arduous, but in a well-managed hospital it is not such as to injure the health. It is found better to give nurses as much work as they can fairly stand with advantage to their bodily well-being, and no more. All hospital managers should take this as their standard. Those who do not should not be hospital managers.

---

THERE has been a falling off in the income of the Great Northern Central Hospital during the past year to a considerable amount. In the face of the very large extension of the institution, this is decidedly unsatisfactory. The new building will be four or five times larger than the old one. How are the increased claims of such an enlarged hospital going to be met? The committee or the officials must bestir themselves, unless they intend the vast agglomeration of bricks and mortar rising in the Holloway Road to be a huge wilderness of deserted corridors and empty wards.

---

MR. JAMES JARDINE presided at the Annual Meeting of the Ancoats Hospital and Dispensary, in the Mayor's parlour on Thursday. Mr. S. Baron, the secretary, read letters of apology from the Bishop of Manchester, the Bishop of Salford, The Right Hon. Sir James Fergusson, M.P., The Right Hon. A. J. Balfour, M.P., Sir W. H. Houldsworth, M.P., Mr. C. E. Schwann, M.P., and others. The medical report and statement of accounts, which presented few special features, were read, and the meeting closed with the usual vote of thanks to the chairman.

A Bacteriological Department has been established in connection with the Berlin Hospital. Professor Virchow is in charge. It is to be hoped the bacteria will duly appreciate the attention which, after waiting so long, they are now receiving at the hands of the distinguished luminaries of medicine and science.

---

The anniversary of the Brabazon Home of Comfort was held at Reigate on Monday. There was an early celebration of Holy Communion in the parish church and service in the afternoon. The sermon was preached by the Rev. A. Dalton, Vicar of All Hallows, Poplar. The offertory amounted to £9 10s. Among the company were the Countess of Meath, the Hon. Victoria Grosvenor, Lady Florence Blunt, Miss Wright, Miss Anderson-Mustead, Miss Saunders, Mrs. Slade, Miss Fitzhugh, Miss Annie Cazenove, Miss Isham, Miss Innes (Lady Superintendent), and others. The proceedings were of a highly interesting character.

---

At the last quarterly meeting of the subscribers to the Grimsby and District Hospital the closed accounts for the year showed a balance due to the Lincoln Bank of £891 on the year's operations. It fortunately happens that a special Jubilee fund, raised during the present year, has reached the substantial sum of £1,600. From this the deficit can be paid off, and £700 will still be left for investment. It would have been much more business-like if the expenditure could have been kept down and the ordinary income increased, thereby enabling the whole of the Jubilee Fund to be invested. Hospitals, like individuals, should be very slow to spend their capital sums. Expenditure, wherever possible, should be met by income, and not by capital. The goose that lays the golden eggs is not difficult to kill.

---

There is a strong feeling among some of the inhabitants of Norwich that their famous hospital is not well served in the way of chaplaincy. There is, it appears, no regular chaplain; but one of the local clergy receives a grant of £50 a year towards the salary of an additional curate, part of whose function it is to do duty at the hospital. Unless there be additional arrangements unknown to us, whereby the patients may receive such religious guidance and comfort as they require, it is not too much to say that there is a condition of positive spiritual destitution at the Norfolk and Norwich Hospital.

---

By the munificence of a donor who does not wish his name to be made public, the sum of £5,000 has been added to an amount of £5,000 already subscribed, making together £10,000, on behalf of the extension fund of the Taunton and Somerset Hospital. By this timely and princely liberality the committee feel that they are free to move forward towards a more complete and efficient scheme of enlargement than they had previously considered possible. The Nursing Institute in connection with the hospital, which was felt to be a heavy burden, will share in this increase of prosperity, to the great comfort and advantage of the nursing staff. The committee of the hospital and the people of Taunton generally express a due sense of their appreciation of the rare generosity of the unknown donor.

During the past year Miss Joss, the nurse appointed by the "Falkirk Association for Providing Trained Nurses," has had charge of 137 new patients, in addition to a considerable number remaining on the list from the previous year. At the annual meeting of this useful Association it was stated that much benefit had resulted to the patients from the skill and kindness of Miss Joss. Many of the patients expressed great gratitude for her unwearied attention, and not a few, by themselves or their friends, promised money donations or other help to the Association.

---

The Annual Meeting of the trustees of the Manchester Royal Infirmary was held on Thursday, Mr. E. S. Heywood presiding. The total number of persons treated during the year 1886-7 was 37,548, at a cost of £22,396, showing a large increase in the number of patients, with a decrease of 10 per cent. in the expenditure. The death-rate had only been 5 per cent.; and the Board expressed its conviction that here, as in all their work, the efforts of the hospital officials had resulted in a very gratifying measure of success. The Earl of Derby was elected president for the ensuing year. The managers of the Infirmary are to be congratulated on having accomplished so much valuable work at such a very considerable diminution of cost.

---

Sir Henry Stephenson, Mayor, presided at the annual meeting of the Sheffield Hospital and Dispensary on Thursday. Mr. J. S. Elvin, Chairman of the Board, Mr. G. F. Lockwood, Master Cutler, Mr. B. Wake, Treasurer, Mr. H. Pawson, Hon. Secretary, and others, were present. There had been, according to the report, a falling off of more than £400 in legacies, causing a small diminution in the total income. The charities of Sheffield during the past year do not seem to have been so well maintained as formerly. We are not aware of any special circumstances to account for this backward progress. What are the working men of Sheffield doing? They earn, at any rate in many departments, large wages. Do they spend them judiciously, or are many of them still given to the disgraceful amusements of dog-fighting, rabbit coursing, pigeon-flying, and other senseless extravagances which used to be so popular in the town and neighbourhood twenty years ago? We fear the working people of this South Yorkshire town are not keeping pace with the times and with other large centres of population in the great northern county. Where are the ministers of religion and the schoolmasters?

---

Eighty-one patients have been sent to the Sandygate Sanatorium from the Sheffield Hospital during the past year, at the sole expense of Mr. Bernard Wake, treasurer to the hospital. Though benevolence may be languishing among the people of Sheffield generally, and self-help may be very feeble among the self-indulgent working-classes, it is clear that Sheffield has at least one generous-hearted and public-spirited citizen. Spurs and whips should not be unfamiliar to any town which is located in the famous horse-county, but Mr. Wake's example should be much more potent than either.

